# A commentary: what do Rebecca Cheptegei and Gisèle Pelicot have in common?

**DOI:** 10.1093/inthealth/ihaf004

**Published:** 2025-02-05

**Authors:** Safieh Shah

**Affiliations:** Institute for Globally Distributed Open Research and Education (IGDORE)

**Keywords:** antenatal care, gender-based violence, intimate partner violence, maternal health, reproductive health, Sustainable Development Goal 5

## Abstract

This commentary accompanies a study that focuses on the profound impact of intimate partner violence (IPV) on women's health in Ethiopia. The study highlights the dose–response relationship between IPV and antenatal care uptake, emphasizing the need for nuanced, context-specific interventions. The commentary highlights the need for new, sustainable and reliable ways of collecting IPV data across countries over time to effectively monitor Sustainable Development Goal 5. It advocates for a multilayered approach, combining healthcare, legal reforms and community-based strategies, to address the social causes of IPV, thereby aiming to critically appraise previously established ways of seeing information and ideas.

Both women were affected by the global crisis of intimate partner violence (IPV), which is closely linked to mass rape, femicide and the worsening state of maternal and neonatal health. This crisis is well-documented in global databases that track violence against women, such as the one maintained by UN Women (https://data.unwomen.org/global-database-on-violence-against-women). On 12 January 2025, while exploring this database, I found that only one in three European countries and one in five African countries have measures for monitoring and evaluating violence against women (VAW) and girls on the aforementioned UN Women website. This lack of measures perpetuates the normalization of IPV at both individual and societal levels, directly fueling these devastating outcomes. This commentary examines the profound impact of IPV on women's health, particularly in Africa. The study highlights how IPV affects antenatal care (ANC) uptake in Ethiopia, emphasizing the need for nuanced, context-specific interventions due to the importance of methodology. Data from the Demographic and Health Surveys (DHS) on IPV encompass >60 developing countries and have been cited in >5000 papers on Google Scholar.^1^

IPV and VAW are both universally condemned, yet they continue to persist, with devastating impacts on women's health globally (https://www.who.int/news-room/fact-sheets/detail/violence-against-women). The prevalence of IPV, particularly in regions like Africa, is a significant contributor to femicide, maternal health risks and poor neonatal outcomes.^2,3^ In 2023, the United Nations reported that Africa had the highest number of femicides, with approximately 21 700 victims, a rate of 2.9 per 100 000 women.^4^ This stark statistic highlights the need to address IPV, particularly within the context of ANC, including perinatal deaths, where its effects are often hidden but are profoundly harmful.^5^

The article ‘Intimate Partner Violence and Antenatal Care Uptake in Ethiopia: A Dose-Response Analysis’ by Bazie Mekonnen Berihun, Abebe Gebremariam, Negussie Deyessa and Cranmer John presents a novel approach to understanding IPV's impact on maternal health. It demonstrates a dose–response relationship between IPV and the uptake of adequate ANC, showing that different forms of IPV—sexual, emotional and physical—have distinct impacts on whether women receive the minimum recommended ≥4 ANC visits (ANC-4). The study used data from Ethiopia's Demographic and Health Survey (EDHS-2016) and applied complex sample logistic regression to predict ANC-4 utilization based on IPV subscales and other predictors such as social, obstetric and empowerment factors. Notably, no previous studies have tested this type of dose–response relationship, marking a significant advancement in IPV scholarship. However, if the DHS IPV data contain substantial, systematic and context-specific biases—as suggested by a study in Nigeria—this could significantly impact the reliability and use of DHS data.^1^

In light of the evidence, this dose–response approach underscores the importance of context-specific data collection. The findings suggest that health interventions should address the various forms and intensities of IPV, as cultural and socioeconomic factors affect both its measurement and impact. By understanding IPV as a spectrum faced, policymakers can better design upstream healthcare interventions tailored to protect the specific needs of pregnant women living with abuse. Another study concluded that improved questions about sexual and psychological IPV need to be fair and consistent across countries and over time for better monitoring of Sustainable Development Goal 5.^6^

The study also highlights a critical intersection: IPV's role in hindering women's access to ANC. Women who experience IPV may avoid seeking care due to fear of their partner's retaliation, control or judgment. This failure to attend ANC increases the risk of maternal complications, including preterm birth, low birth weight and mental health issues.^7^ By integrating IPV screening into ANC services, healthcare professionals can identify at-risk women early and provide the support necessary to prevent further IPV.

This research paves the way for new policy development and interdisciplinary collaboration by highlighting the need to rethink current IPV measurement and intervention models. These findings emphasize the need for a multilayered approach to address IPV by targeting males and combining healthcare, legal reforms and community-based strategies.

In conclusion, the study offers a critical advancement in understanding IPV's impact on maternal health. By shifting away from binary data models and embracing a more nuanced understanding of IPV's effects, this research provides a roadmap for more targeted and effective interventions. As IPV remains a leading cause of femicide and maternal morbidity worldwide, this work offers vital insights that can inform future research and policy to reduce the global burden of IPV by addressing this social problem that males perpetrate, thus improving the health and safety of girls and women everywhere.

## Data Availability

No new data were generated or analyzed in support of this commentary.
